# Integrated metagenomic and culturomic strategies to mine and validate beneficial rhizosphere *Actinobacteria* from lavender

**DOI:** 10.3389/fpls.2026.1745076

**Published:** 2026-02-19

**Authors:** Xugela Habuding, Jiayi Chen, Jinfang Zhu, Guanru Wang, Lan Ma, Tajiguli Abulikemu

**Affiliations:** 1 Xinjiang Key Laboratory of Special Species Conservation and Regulatory Biology, College of Life Science, Xinjiang Normal University, Urumqi, China; 2 College of Food Science and Pharmacy, Xinjiang Agricultural University, Urumqi, China

**Keywords:** actinobacteria, lavender, microorganisms, plant growth-promoting rhizobacteria (PGPR), rhizosphere soil, saline-alkali tolerance

## Abstract

**Introduction:**

The lavender industry faces significant constraints from soil salinization and continuous cropping obstacles. However, systematic exploration and functional analysis of beneficial rhizosphere microbial resources, particularly Actinobacteria, remain inadequate.

**Methods:**

To address this, we integrated metagenomic and culturomic strategies to investigate the rhizosphere and endophytic microbiomes in saline-alkaline lavender cultivation areas in Huocheng, China (soil pH ~8.04, salt ~0.074%). Metagenomic functional annotation and soil factor correlation analysis guided a subsequent culturomics approach to isolate strains. Isolates were screened for plant growth-promoting (PGP) traits, and selected strains were evaluated in pot inoculation experiments with Arabidopsis thaliana.

**Results:**

High-throughput sequencing revealed that Actinomycetota dominated the microbial communities, with Streptomyces and Nocardioides as key genera. Metagenomic analysis showed the community was enriched with functional genes related to saline-alkaline stress response, secondary metabolite synthesis, and nutrient cycling, whose distribution correlated significantly with soil pH and salinity. From this resource, 10 actinobacterial strains with multiple PGP traits (e.g., P-solubilization, siderophore production, IAA, ACC deaminase, and nitrogenase activity) were obtained. Pot experiments confirmed that these saline-alkaline-derived actinobacteria, both as single strains and as a bacterial consortium (C4 + A1), significantly promoted the growth of A. thaliana.

**Discussion:**

This study achieves a closed-loop verification from in silico functional prediction to empirical validation of beneficial strains. It provides the first systematic elucidation of the functional adaptation mechanisms of the lavender rhizosphere actinobacterial community under saline-alkaline stress and identifies elite microbial resources with both stress tolerance and PGP functions. The findings offer novel microbial agents and a theoretical foundation for developing specialized inoculants to mitigate saline-alkaline obstacles in lavender cultivation.

## 1 Introduction

The long-term overuse of chemical fertilizers and pesticides in agriculture has increased crop yields at the cost of soil degradation, environmental pollution, and impaired ecosystem functioning (Yu et al., 2024). To achieve sustainable agriculture, harnessing beneficial microbial resources from the rhizosphere and plant endophytic environments has become a crucial pathway for advancing the transition to green agriculture (Bukhat et al., 2020; Behera et al., 2024). The mutualistic symbiosis between plants and microorganisms holds significant potential for promoting growth, enhancing stress resistance, and improving soil health (Bukhat et al., 2020; Asghar et al., 2024). Systematically elucidating the mechanisms of plant-microbe interactions and screening strains with defined probiotic functions lay a theoretical foundation for developing novel microbial fertilizers (Xun et al., 2021; Wang et al., 2022; Asghar et al., 2024).

As a central element of the plant micro-ecosystem, the structure and function of the plant rhizosphere microbiome are defined by a combination of plant genotype, developmental stage, soil type, and environmental factors (Jia et al., 2020; Liu et al., 2020; Shankar et al., 2023). Rhizosphere microbes orchestrate processes ranging from nutrient cycling to soil-borne pathogen suppression. Furthermore, their community assemblies display marked tissue specificity (e.g., root, stem, leaf) and spatial partitioning (e.g., rhizosphere soil versus endophytic compartments) (Yuan et al., 2022). Importantly, the rhizosphere constitutes a pivotal hotspot for plant-driven microbial recruitment through root exudates. These compounds facilitate the selective enrichment of microbial cohorts with particular functions, which in turn orchestrate the assembly of the endophytic community (Park et al., 2023; Cao et al., 2025). The endophytic microbiota is commonly conceptualized as a “functionally specialized subset” of the rhizosphere microbiome, with the assembly of both being intrinsically linked by a root exudate-mediated recruitment mechanism (Singh et al., 2004; Tan et al., 2017). For example, research into continuous cropping obstacles has shown that enriched soil-borne pathogens in the rhizosphere can colonize the roots, which in turn causes a marked depletion of endophytic microbial diversity (Tan et al., 2017). Therefore, concurrently profiling both the rhizosphere soil and plant endophytic microbial communities is of strategic importance for elucidating the mechanisms of micro-ecological imbalance and for mining highly effective strains with antagonistic and plant growth-promoting capabilities.

Within the diverse community of rhizosphere microorganisms, *Actinomycetota* stand out for their extensive metabolic diversity, positioning them as prominent resources for promoting plant growth and enabling biological control (Borah et al., 2022). This group of microorganisms can not only secrete siderophores, solubilize insoluble phosphate, and produce 1-Aminocyclopropane-1-carboxylic acid (ACC) deaminase to alleviate environmental stress and promote plant growth, but also synthesize antimicrobial secondary metabolites (e.g., antibiotics) to effectively suppress pathogen growth (Bilyk and Luzhetskyy, 2016; Long et al., 2022; Sivagnanam et al., 2022). In recent years, the development of high-throughput technologies, such as metagenomics, has enabled the systematic dissection of plant-microbe interaction networks, providing a powerful tool for function-driven mining of microbial resources (Zhang et al., 2019).

Lavender is a high-value economic crop, with over 90% of its cultivation area in China located in the Ili region of Xinjiang (Ren et al., 2008). It is widely used in the perfume (Batiha et al., 2023), essential oil (Alves-Silva et al., 2023), and pharmaceutical industries (Kulabas et al., 2018; Héral et al., 2020). Particularly, the narrow-leaved lavender (*Lavandula angustifolia* Mill., LAM) and the broad-leaved lavender (*Lavandula latifolia* Vill., LLV) represent two commercially important varieties (Li et al., 2023). However, lavender cultivation frequently faces challenges such as replant problems, soil salinization, and frequent disease outbreaks, which severely constrain the sustainable development of the industry. Furthermore, current research on the structure and function of the lavender root microbiome remains limited, with a particular lack of systematic exploration of rhizosphere plant growth-promoting bacteria and their underlying mechanisms. Previous studies have shown that continuous cropping obstacles significantly reduce the diversity of both rhizosphere soil and endophytic microbiota, while leading to the enrichment of pathogenic fungi such as *Leotiomycetes* and *Mycocentrospora*, suggesting that microbial community dysbiosis is a key contributor to the challenges associated with monoculture (Tan et al., 2017). Therefore, integrating the study of both the rhizosphere soil and endophytic microbiota of lavender constitutes a key entry point for deciphering its micro-ecological imbalance and screening for probiotic strains.

In recent years, strategies integrating metagenomics with culturomics have provided powerful support for a comprehensive understanding of plant-microbe interactions (Jagadesh et al., 2024; Wani et al., 2025). High-throughput sequencing can systematically reveal the composition and functional potential of microbial communities, whereas culturomics enables the isolation of active strains and their subsequent functional validation (Cui et al., 2023). Notably, the rhizosphere microbiome serves not only as a significant reservoir for endophytes but also as a critical interface responding to environmental changes and plant physiological status (Park et al., 2023; Asiloglu et al., 2024).

However, current knowledge regarding the structure and function of the lavender root microbiome remains limited, particularly lacking a systematic exploration of beneficial rhizosphere actinobacterial resources and an integrated elucidation of their growth-promoting and stress-resistance mechanisms. Therefore, this study aimed to integrate metagenomic and culturomic strategies, using both LLV and LAM as research subjects. First, we systematically analyzed the composition and diversity of microbial communities in root, stem, and leaf tissues as well as rhizosphere soil to identify dominant taxa. Subsequently, focusing on the rhizosphere soil, we directionally mined beneficial actinobacterial resources with plant growth-promoting and stress-tolerant traits, clarifying their community distribution and ecological functions. Finally, through functional validation and inoculation experiments, elite strains were screened. This study not only provides a theoretical basis for clarifying the regulatory mechanisms of the lavender rhizosphere microecology but also offers important microbial resources and scientific support for developing efficient microbial inoculants and promoting the ecological cultivation and sustainable development of the lavender industry.

## 2 Materials and methods

### 2.1 Sample collection and processing

Plant and soil samples were collected on June 20, 2023, from a healthy, continuously cropped Lavender field in Huocheng County, Xinjiang, China (80°40′44″ E, 43°39′08″ N, altitude ~780 m). The weather was overcast during sampling, with an air temperature of 20-31°C (data sourced from the China Meteorological Administration). LLV and LAM were selected as the study subjects. 5 sampling points were established for each variety. At each point, 3 plants with uniform and vigorous growth were selected. Root, stem, and leaf tissues were sampled concurrently. For rhizosphere soil collection, roots at approximately 30 cm below the soil surface were carefully excavated, and the soil adhering to the root surface was gently brushed off using a sterile brush. The rhizosphere soil samples collected from multiple sampling points for each lavender variety were thoroughly mixed to form a composite sample, which was then evenly divided into three replicates. One for metagenomic sequencing, one for soil physicochemical property analysis, and one for microbial isolation. All samples were immediately placed on ice and transported to the laboratory. Soil aliquots designated for DNA extraction were stored at -80°C, while those intended for physicochemical analysis and microbial isolation were air-dried and stored for subsequent use. Following this procedure, a total of 6 rhizosphere soil samples were obtained-3 for LLV and 3 for LAM.

### 2.2 Analysis of rhizosphere soil physicochemical properties

Rhizosphere soil samples were sent to the Eco-Environment Analysis and Testing Center of the Xinjiang Institute of Ecology and Geography, Chinese Academy of Sciences, for the determination of the following indicators: total salt content, pH, organic matter, total nitrogen, available nitrogen, and available phosphorus.

### 2.3 Metagenomic sequencing

#### 2.3.1 Amplicon sequencing for taxonomic analysis of microbial communities

Total DNA was extracted from the rhizosphere soil microorganisms and the endophytic bacteria of the roots, stems, and leaves using a commercial DNA extraction kit (purchased from Thermo Fisher Scientific (China) Co., Ltd.). The V4-V5 hypervariable region of the bacterial 16S rRNA gene was amplified using primers 515F (5’-GTGCCAGCMGCCGCGG-3’) and 907R (5’-CCGTCAATTCMTTTRAGTTT-3’). The fungal ITS region was amplified using primers ITS1F and ITS2R. The PCR reaction system utilized 2×Easy Taq PCR Super Mix (TransGen Biotech, Beijing, China). DNA Marker was obtained from Sangon Biotech (Shanghai, China), and agarose was obtained from GENE COMPANY LTD (China). The amplified products were purified, and libraries were constructed. Sequencing was performed on the Illumina HiSeq 4000 platform with a PE150 strategy.

Raw data were subjected to quality control and denoising. Operational Taxonomic Units (OTUs) were clustered at 97% similarity using USEARCH, and taxonomic annotation was performed against the RDP and UNITE databases. Alpha diversity indices (Chao1, Shannon, Simpson) and beta diversity analyses (PCoA, NMDS) were conducted using QIIME2 and the R language.

#### 2.3.2 Shotgun metagenomic sequencing for functional gene profiling

Metagenomic sequencing employed a shotgun strategy, and functional gene annotation was based on the KEGG, COG, and CAZy databases. The association between microbial functions and soil factors was analyzed using Redundancy Analysis (RDA) and the Mantel test.

### 2.4 Isolation, purification and identification of rhizosphere *Actinobacteria*

*Actinobacteria* were isolated using a modified Gauze’s No. 1 medium. Soil samples were subjected to a 10-fold serial dilution, spread onto the agar plates, and incubated at 37c°C for 7–14 days. Distinct colonies were selected and repeatedly streaked for purification. Preliminary identification was based on colony morphology, Gram staining, and spore structure.

Genomic DNA was extracted using the Chelex-100 method (reagent purchased from Bio-Rad, USA). The nearly full-length 16S rRNA gene was amplified with universal primers 27F and 1492R. The PCR products were sequenced by Sangon Biotech (Shanghai, China). The resulting sequences were compared against the EzBioCloud database for taxonomic assignment, and a phylogenetic tree was constructed using MEGA software (version 12.0).

Note: Throughout this manuscript, the term “actinobacteria” refers to the bacterial isolates that were preliminarily selected based on colony and mycelial morphology characteristic of filamentous actinomycetes, and taxonomically confirmed by 16S rRNA gene sequencing to belong to the phylum *Actinobacteria* (synonym *Actinomycetota*).

### 2.5 Determination of salt-alkali tolerance and plant growth-promoting traits of *Actinobacteria*

Salt and Alkali Tolerance: Bacterial growth was assessed across media containing 2% to 25% (w/v) NaCl and adjusted to pH levels ranging from 5 to 12 to evaluate their salt and alkali tolerance, respectively.

Nitrogenase (NITS) Activity: NITS activity was measured using an ELISA kit (Jiangsu Enzyme Immunity Industrial Co., Ltd., China) according to the manufacturer’s instructions. Activity was calculated based on a standard curve (0–120 U/L) and expressed in units per liter (U/L).

ACC Deaminase Activity: ACC deaminase activity was determined using an ELISA kit (Jiangsu Enzyme Immunity Industrial Co., Ltd., China) as per the protocol. Activity was calculated using a standard curve (0–48 U/L) and reported in U/L.

Siderophore Production: Siderophore production was preliminarily evaluated using the Chrome Azurol S (CAS) plate assay and further quantified via the liquid CAS assay after cultivation, with results expressed as the A/Ar ratio. The CAS medium used was purchased from Thermo Fisher Scientific (China).

Phosphate Solubilization: The phosphate-solubilizing ability was initially screened on solid NBRIP medium by measuring the dissolution zone after 7 days of incubation. Quantitative analysis was performed in liquid culture using the molybdenum blue colorimetric method to determine the concentration of soluble phosphorus. All chemical reagents used, including potassium hydroxide, sodium molybdate, concentrated sulfuric acid, and phosphorus standard solution (analytical grade), were purchased from Zhiyuan Chemical Reagent Co., Ltd. (Tianjin, China).

IAA Production: The ability to produce indole-3-acetic acid (IAA) was assessed by growing strains in LB broth supplemented with L-tryptophan. The culture supernatant was mixed with Salkowski reagent, and the absorbance was measured at 530 nm. IAA concentration was quantified using a standard curve.

### 2.6 Preparation of actinobacterial inoculum for pot experiment

For inoculum preparation, each strain preserved on agar slants (containing glucose 4 g/L, yeast extract 4 g/L, malt extract 5 g/L, peptone 4 g/L, NaCl 10 g/L, and agar 15 g/L, pH 7.5) was first activated on fresh solid medium. After incubation at 37 °C for 24-48 h, a single colony was picked with a sterile loop and inoculated into a 50 mL flask containing 10 mL of liquid seed medium. The culture was then incubated at 37 °C with shaking at 180 rpm until the optical density at 600 nm (OD_600_) reached approximately 0.6. This seed culture was used to inoculate (1 % v/v) 300 mL of production medium in a 500 mL flask, which was incubated under the same conditions until the late-exponential phase. After cultivation, cells were harvested by centrifugation at 8000 × g for 10 min at 4 °C using a centrifuge (LEGEND MICRO 17, Thermo Fisher Scientific, China). The pellet was then washed twice gently with sterile saline or phosphate buffer to remove residual medium components. The suspension was adjusted to a final OD_600_ of 0.5 ± 0.04, which served as the single-strain inoculum. For co-inoculation treatments (e.g., *Microbacterium algeriense* C4 + *Streptomyces* sp. A1), equal volumes of the respective single-strain suspensions were mixed thoroughly. All inoculants were prepared freshly and used within 4 h of preparation.

### 2.7 Pot experiment with *A*. *thaliana* inoculation

Four strains (*Streptomyces* sp. A1, *Streptomyces marianii* B3, *Streptomyces marianii*C3, *Microbacterium algeriense* C4) showing prominent plant growth−promoting traits were selected for single-strain and co-inoculation (C4 + A1) treatments. *A*. *thaliana* (ecotype Col-0) seeds were surface-sterilized and sown on solid MS medium. After stratification at 4 °C for 2 days, plates were transferred to a growth chamber set at 22 °C with a 16 h light/8 h dark photoperiod for germination and early seedling growth. At the four-leaf stage, seedlings were transplanted into pots (10.5 cm in diameter × 11 cm in height) filled with a mixed substrate consisting of vermiculite, perlite, and native lavender rhizosphere soil (3:1:1, v/v/v). After a 3-day acclimatization period, plants were inoculated by applying 1 mL of bacterial suspension (OD_600_ = 0.5 ± 0.04) evenly around the root zone; control plants received the same volume of sterile water. Following inoculation, all plants were cultivated in the same growth chamber. After 60 days, plants were harvested to measure growth and physiological parameters, including plant height, root length, biomass, leaf number, and chlorophyll content.

### 2.8 Determination of physiological and biochemical parameters in *A*. *thaliana*

After 60 days of cultivation, 10–15 seedlings were randomly selected from each treatment group. The seedlings were rinsed with distilled water and gently blotted dry before weighing to record the fresh weight (g). The roots were separately weighed to record the root fresh weight (g). Subsequently, the seedlings were placed in a drying oven at 75°C for 24 hours and then weighed again to determine the dry weight (g).

For other randomly selected seedlings from different treatment groups, plant height (cm) and root length (cm) were measured using a vernier caliper. Leaf area was determined using a leaf area meter.

To determine chlorophyll content, 0.2 g of fresh leaf sample from different treatment groups was placed in a stoppered graduated test tube. Then, 20 ml of a 1:1 (v/v) acetone and absolute ethanol extraction solution was added. After light-proof treatment, the mixture was kept in the dark for extraction. When the sample in the tube turned completely white (after approximately 24 hours), the extract was filtered into a brown volumetric flask. The absorbance of the extract was measured at wavelengths of 645 nm, 663 nm, and 652 nm using a UV-Vis spectrophotometer, with the acetone-ethanol mixture serving as the blank control.

The contents of chlorophyll a, chlorophyll b, and total chlorophyll (mg·L^-1^) were calculated using the following formulas:


$${\text{C}}_{a}=12.72\times {\text{A}}_{663}-2.59\times {\text{A}}_{645}\left(\text{Chlorophyll a content}\right)$$

$${\text{C}}_{b}=22.88\times {\text{A}}_{645}-4.67\times {\text{A}}_{663}\left(\text{Chlorophyll b content}\right)$$

$$\text{Cr}=\;\frac{{\text{A}}_{652}\times 1000}{34.5}\;\left(\text{Total Chlorophyll Content}\right)$$

### 2.9 Data processing and analysis

All experiments were performed with at least three independent replicates. Data are expressed as the mean ± standard deviation. Statistical analysis was conducted using SPSS software (version 26.0). Differences between treatments were assessed by one-way analysis of variance (ANOVA), followed by Duncan’s multiple range test for *post-hoc* comparisons. Statistical significance was defined at ^*^p^*^< 0.05, and different lowercase letters in figures/tables indicate significant differences between groups. Graphs were generated using GraphPad Prism (version 9).

## 3 Results

### 3.1 Structure and diversity of endophytic bacterial communities in lavender roots, stems, and leaves

**Figure 1:**
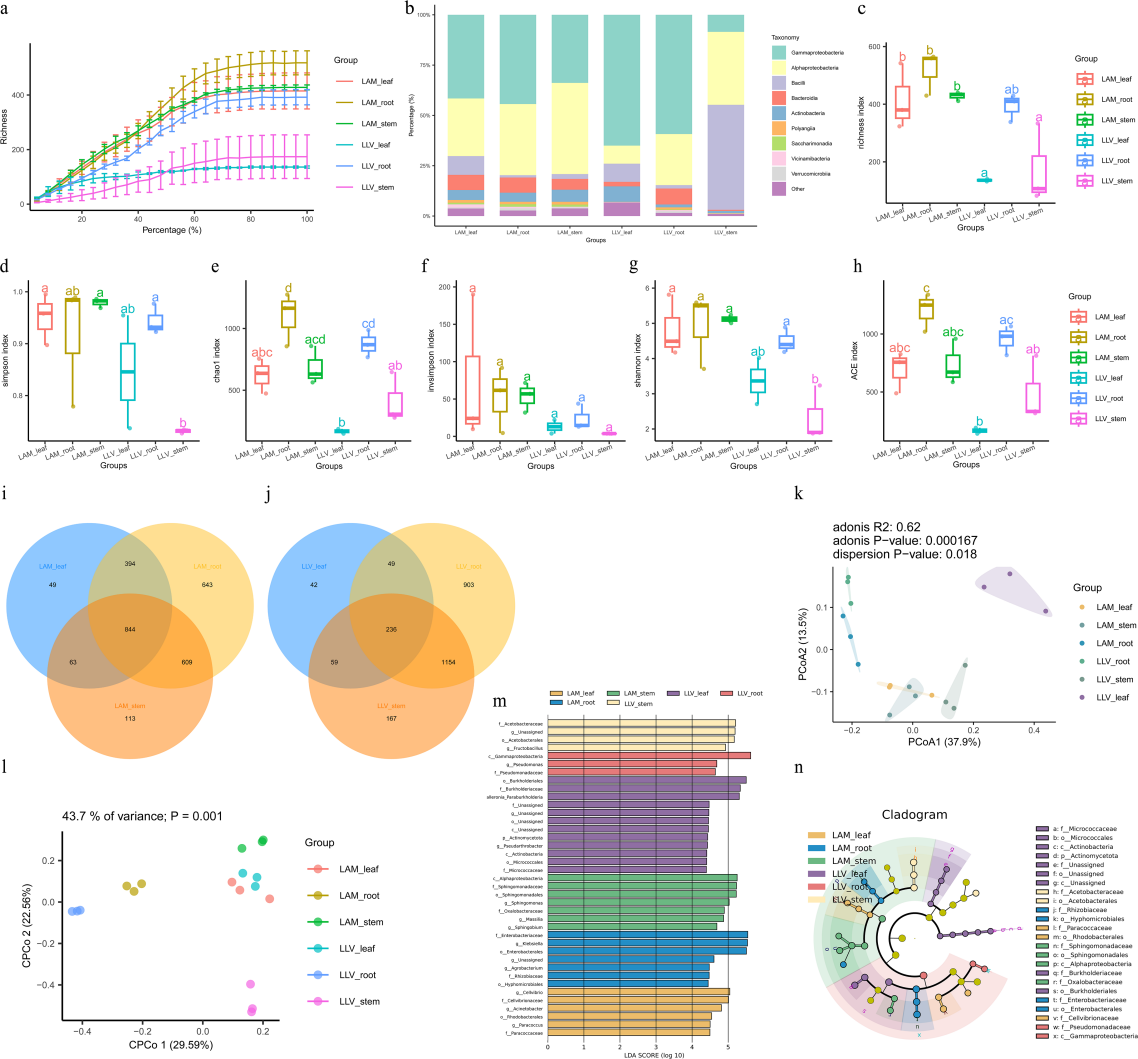
Structure and diversity of endophytic bacterial communities in roots, stems, and leaves of *Lavandula latifolia* Vill. and *Lavandula angustifolia* Mill. **(a)** Rarefaction curves of 16S rRNA gene sequences. **(b)** Relative abundance of dominant bacterial phyla in roots, stems, and leaves of *Lavandula latifolia* Vill. (LLV) and *Lavandula angustifolia* Mill. (LAM). **(c-h)** Alpha diversity indices (Chao1, ACE, Shannon, Simpson, Inverse Simpson) across tissues. Different lowercase letters indicate significant differences (p< 0.05, one-way ANOVA with Duncan’s test, n = 5). **(i, j)** Venn diagrams of unique and shared ASVs among tissues for LAM **(i)** and LLV **(j)**. **(k)** PCoA plot based on Bray-Curtis distances, showing community separation by tissue. Structure and diversity of endophytic bacterial communities in roots, stems, and leaves of *Lavandula latifolia* Vill. and *Lavandula angustifolia* Mill. **(l)** CPCoA plot corroborating tissue effect (P = 0.001). **(m)** LEfSe analysis identifying tissue-enriched bacterial taxa (LDA score > 4.0). **(n)** Cladogram of enriched taxa from **(m)**. Roots showed the highest diversity and enrichment of *Actinomycetota*.

**Figure 2:**
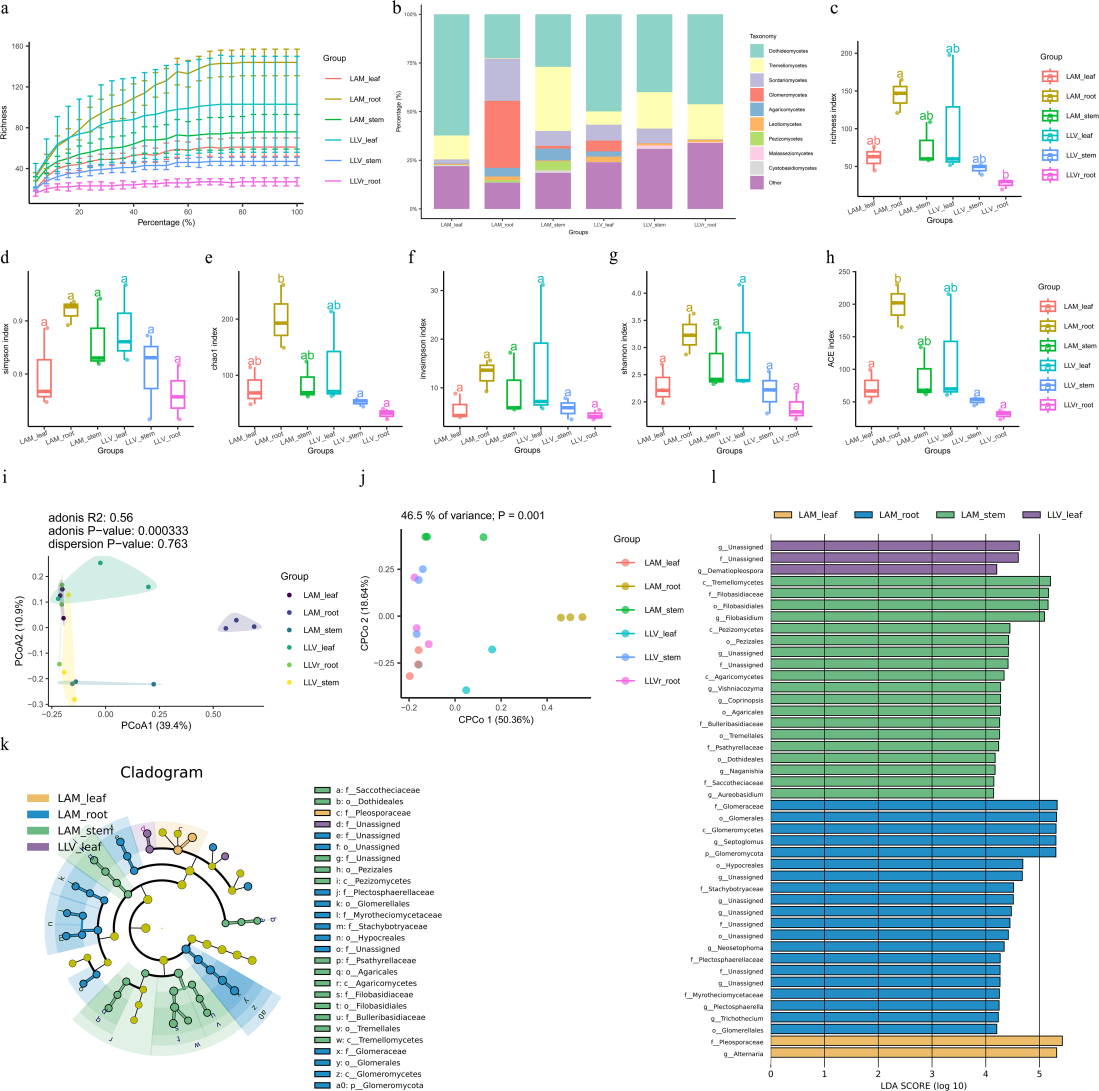
Structure and diversity of endophytic fungal communities in roots, stems, and leaves of *Lavandula latifolia* Vill. and *Lavandula angustifolia* Mill. **(a)** Rarefaction curves of fungal ITS sequences, indicating sufficient sequencing depth. **(b)** Relative abundance of dominant fungal phyla in roots, stems, and leaves of LLV and LAM. **(c-h)** Alpha diversity indices (Chao1, ACE, Shannon, Simpson, Inverse Simpson) across tissues. Different lowercase letters indicate significant differences (p< 0.05, one-way ANOVA with Duncan’s test, n = 5). **(i)** PCoA plot based on Bray-Curtis distances, showing distinct clustering by tissue. **(j)** CPCoA plot further confirming the tissue effect (P = 0.001). Structure and diversity of endophytic fungal communities in roots, stems, and leaves of *Lavandula latifolia* Vill. and *Lavandula angustifolia* Mill. **(k)** Cladogram from LEfSe analysis showing the phylogenetic distribution of tissue-enriched fungal taxa. **(l)** Histogram of LDA scores (LDA > 4) for the significantly enriched fungal taxa in each tissue. Fungal communities exhibited clear tissue specificity, with roots harboring the highest diversity and enrichment of soil-derived taxa like *Mortierellomycota*.

To characterize the structure and diversity of endophytic microbial communities, high-throughput sequencing targeting the bacterial 16S rRNA gene and fungal ITS region was performed on root, stem, and leaf tissues of LLV and LAM. The rarefaction curves for all samples plateaued, confirming adequate sequencing coverage for comprehensive microbial analysis (**Figures 1a**; **2a**).

#### 3.1.1 Bacterial community structure and diversity

Alpha diversity analysis revealed that the bacterial communities in the roots exhibited significantly higher richness (Chao1 and ACE indices) and diversity (Shannon index) compared to those in the stems and leaves (p< 0.05). The leaf tissues hosted the lowest bacterial diversity, indicating a strong filtering effect of plant tissue niches on bacterial community assembly (**Figures 1c–h**). Principal Coordinates Analysis (PCoA) based on Bray-Curtis distances further demonstrated a clear separation in bacterial community structure among the root, stem, and leaf tissues (ANOSIM, R^2^ = 0.62, p = 0.000167), confirming that the ecological niche is a key driver of bacterial community composition (**Figures 1k, l**). Venn diagrams illustrated that the number of core Amplicon Sequence Variants (ASVs) shared among the root, stem, and leaf niches was low. Instead, unique ASVs dominated each specific tissue, with the root compartment exhibiting the highest number of unique ASVs (**Figures 1i, j**). At the phylum level, *Pseudomonadota*, *Bacillota*, *Bacteroidota*, and *Actinomycetota* were identified as the dominant bacterial phyla (**Figure 1b**). Linear Discriminant Analysis Effect Size (LEfSe) identified several bacterial biomarkers that were significantly enriched in the different lavender tissues (LDA score > 4.0). For instance, the phylum *Actinomycetota* and its subordinate taxa, such as the family *Micrococaceae* and the order *Micrococales*, were significantly enriched in the roots. In contrast, the stems and leaves were enriched with different lineages within the *Pseudomonadota*, including families such as *Acetobacteraceae*, *Burkholderiaceae*, and *Pseudomonadaceae* (**Figures 1m, n**).

#### 3.1.2 Fungal community structure and diversity

Analysis of the fungal communities (**Figure 2**) revealed a tissue-specific pattern similar to that of the bacteria. Alpha diversity analysis indicated that the richness and diversity of fungal communities in the roots were also significantly higher than those in other tissues (**Figures 2b–h**). Principal Coordinates Analysis (PCoA) based on Bray-Curtis distances showed significant differences in fungal community structure among the different plant tissues (ADONIS, R^2^ = 0.56, p = 0.000333). Constrained Principal Coordinates Analysis (CPCoA) further confirmed the significant influence of tissue type on fungal community composition (P = 0.001) (**Figures 2i, j**). The cladogram and LEfSe analysis further identified distinct tissue-specific enrichments of fungal taxa. The leaves were predominantly enriched with taxa related to the phylum Ascomycota, while the roots showed significant enrichment of soil-derived fungal groups such as Mortierellomycota (**Figures 2k, l**).

In summary, the endophytic microbial communities in lavender roots, stems, and leaves, for both bacteria and fungi, exhibited significant differences and strong tissue specificity. The root was the niche with the highest microbial diversity, and the significant enrichment of key bacterial phyla like Actinomycetota in the roots suggests their potential important role in plant-microbe interactions.

### 3.2 Association between rhizosphere microbial community structure and functional traits in lavender

**Figure 3:**
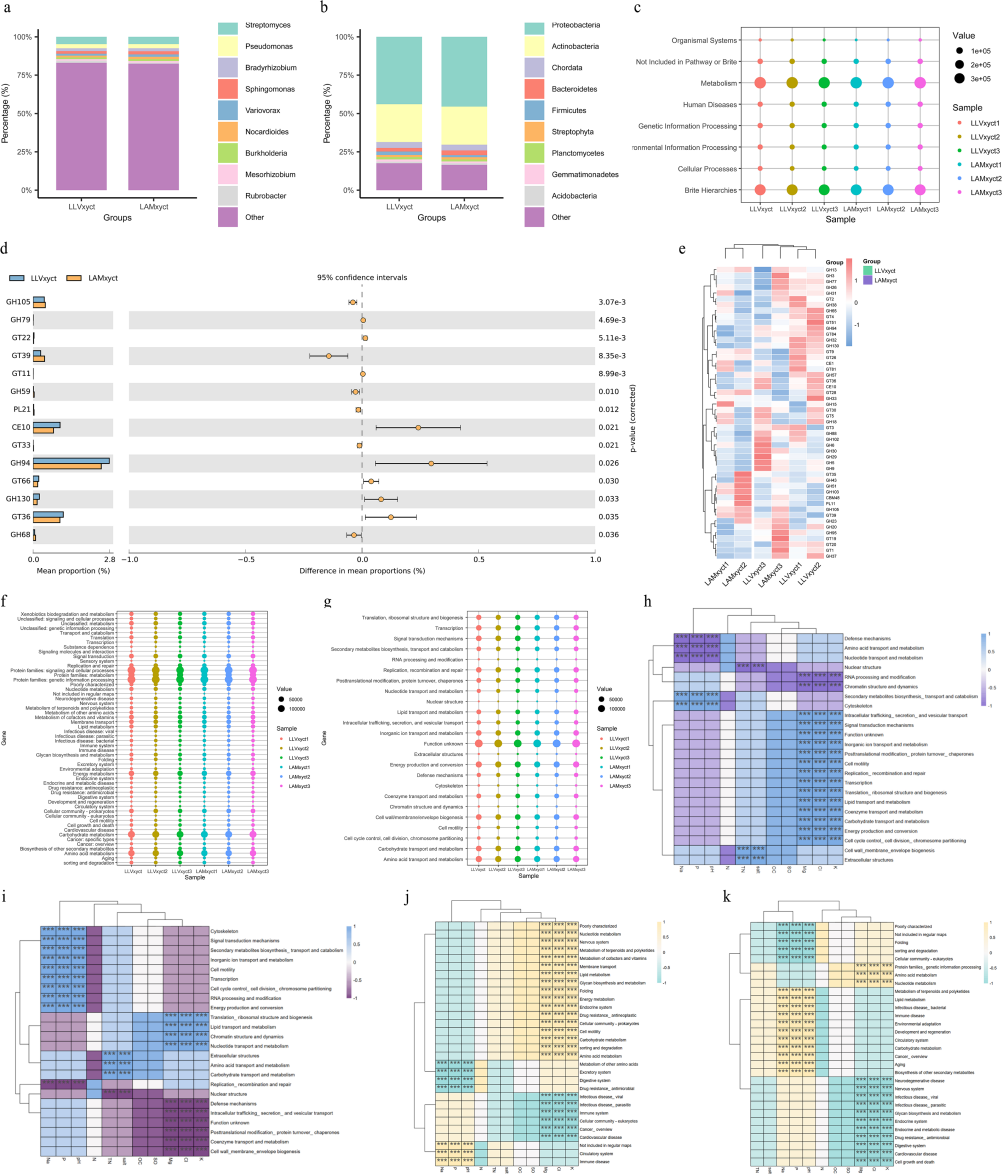
Composition and functional potential of the rhizosphere bacterial community in two lavender species. **(a, b)** Relative abundance of rhizosphere bacteria at the **(a)** phylum and **(b)** genus level in LLV and LAM. **(c, f)** KEGG functional annotation of shotgun metagenomic sequences at **(c)** Level 1 and **(f)** Level 2 categories. **(d, e)** Profile of carbohydrate-active enzymes (CAZymes): **(d)** abundance of the top 50 CAZy families and **(e)** differential abundance between lavender species. **(g)** COG functional classification at Level 2, comparing the two species. Composition and functional potential of the rhizosphere bacterial community in two lavender species. **(h, i)** Redundancy analysis (RDA) biplots showing the correlation between soil physicochemical properties (e.g., pH, AP, SOM) and COG functional categories in **(h)** LAM and **(i)** LLV (p< 0.05). **(j, k)** RDA biplots showing the correlation between soil properties and KEGG Level 2 metabolic pathways in **(j)** LAM and **(k)** LLV. The community was dominated by *Actinomycetota*, with functional genes enriched in metabolism, stress response, and nutrient cycling, showing significant correlations with soil factors.

Analysis of the rhizosphere soil microbial community composition in lavender revealed that at the phylum level, *Actinomycetota* was the absolutely dominant phylum, followed by *Pseudomonadota* and *Bacillota* (**Figure 3a**). At the genus level, the relative abundances of *Streptomyces* and *Nocardioides* were significantly higher than those of other taxa, indicating their core status in the lavender rhizosphere micro-ecosystem (**Figure 3b**).

Functional annotation results showed that in the KEGG Level 1 classification, microbial gene functions were primarily enriched in major categories such as “Metabolism”, “Genetic Information Processing”, and “Environmental Information Processing” (**Figure 3c**). Further subdivision of the “Metabolism” pathway at Level 2 revealed high enrichment of functions closely related to plant nutrient uptake and stress response, including “Carbohydrate metabolism”, “Amino acid metabolism”, and “Metabolism of cofactors and vitamins” (**Figure 3f**). Notably, the “Metabolism of terpenoids and polyketides” pathway also exhibited relatively high abundance, suggesting that rhizosphere microorganisms may be involved in the synthesis or regulation of plant secondary metabolites, thereby indirectly enhancing plant stress resistance.

Annotation results from the CAZy database indicated that Glycoside Hydrolases (GHs) and Glycosyl Transferases (GTs) were the two most abundant carbohydrate-active enzyme families, implying that this microbial community plays a significant role in the decomposition of complex carbohydrates like cellulose and hemicellulose, thereby facilitating soil organic matter degradation and nutrient cycling.

CAZy database annotation revealed that Glycoside Hydrolases (GHs) and Glycosyl Transferases (GTs) were the two most abundant carbohydrate-active enzyme (CAZyme) families. Based on this genetic potential, we speculate that this microbial community may play an important role in decomposing complex carbohydrates, such as cellulose and hemicellulose, thereby potentially contributing to soil organic matter degradation and nutrient cycling (**Figures 3d, e**).

COG functional classification analysis further revealed significant enrichment of genes associated with stress response and competitive adaptation, primarily including “Transcription”, “Replication, recombination and repair”, “Defense mechanisms”, and “Secondary metabolites biosynthesis, transport and catabolism” (**Figure 3g**). These genomic features suggest that the microbial community may possess the genetic underpinnings to adapt to the saline-alkaline stress environment in Xinjiang and to potentially synthesize antimicrobial and stress-resistance compounds.

Redundancy analysis (RDA) results demonstrated significant correlations (p< 0.05) between key soil physicochemical factors (e.g., pH, available phosphorus AP, soil organic matter SOM) and the distribution of various COG functional categories (e.g., amino acid transport and metabolism, carbohydrate transport and metabolism) as well as KEGG Level 2 metabolic pathways (e.g., nitrogen metabolism, sulfur metabolism, ABC transporters). This finding suggests that the unique soil environment in the lavender rhizosphere may have selected for and shaped a functionally active microbial community. This community, through mechanisms such as efficient nutrient cycling, stress response, and secondary metabolite synthesis, could potentially contribute to improving the rhizosphere microecology and enhancing plant stress tolerance (**Figures 3h–k**).

### 3.3 Isolation and identification of plant growth-promoting *Actinobacteria* from the lavender rhizosphere

A total of 168 actinobacterial strains were isolated from 6 lavender rhizosphere soil samples using 4 different isolation media: Gauze’s No. 1 agar medium, inorganic phosphate-solubilizing bacterium medium, organic phosphate-solubilizing bacterium medium, and nitrogen-fixing bacterium medium. Based on colony morphology (size, shape, color, margin features) and microscopic observation of mycelial structure, 30 representative strains were initially selected. Phylogenetic analysis of their 16S rDNA sequences revealed that these strains belonged to 4 genera: *Streptomyces*, *Microbacterium*, *Micrococcus*, and *Nocardiopsis*. Among them, *Streptomyces* was the dominant genus, comprising 22 strains, followed by *Microbacterium* (4 strains), while *Micrococcus* and *Nocardiopsis* each represented by 2 strains.

**Figure 4:**
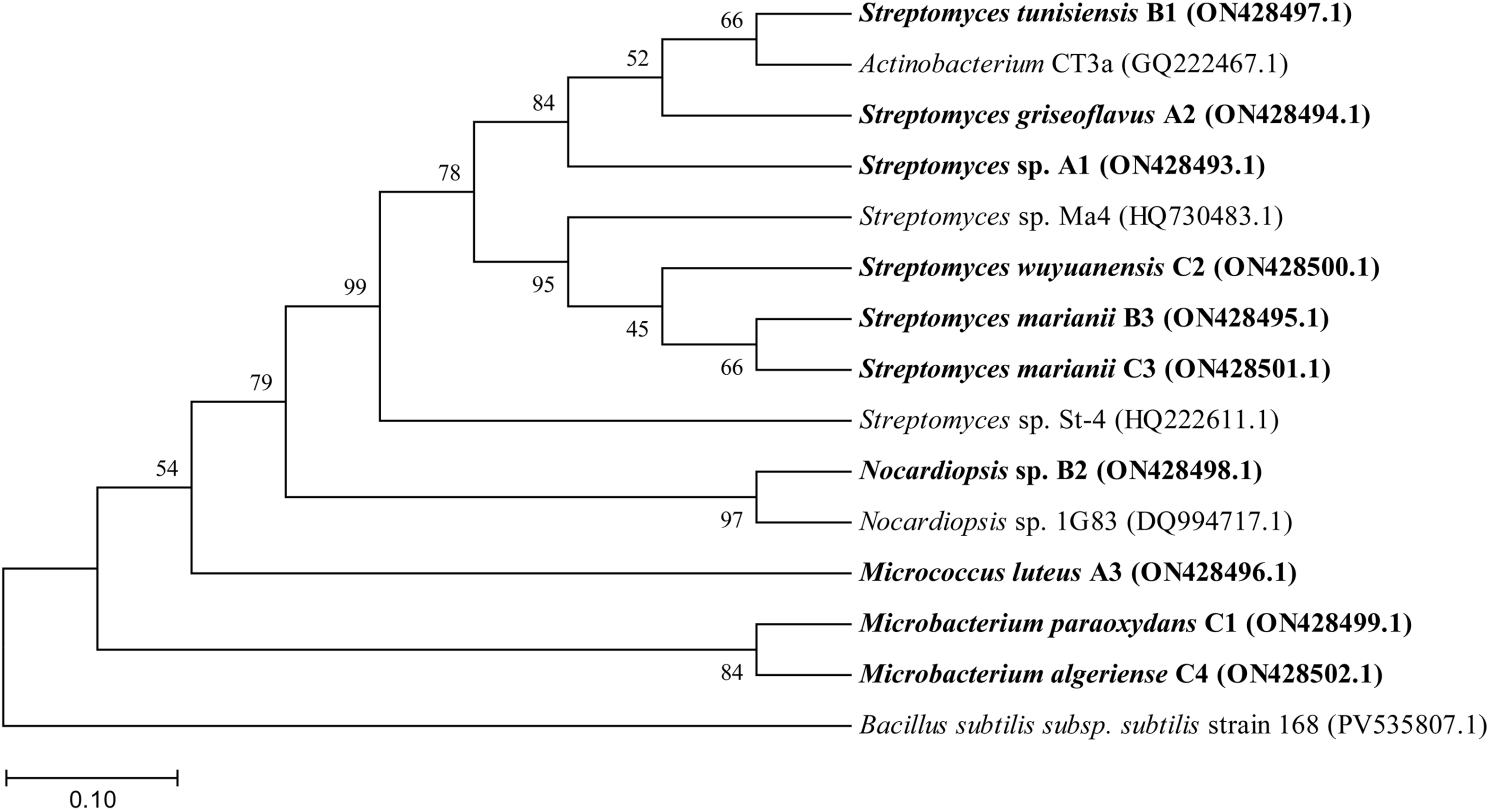
Phylogenetic relationships of 10 actinobacterial strains isolated from the lavender rhizosphere. Maximum-likelihood phylogenetic tree based on nearly full-length 16S rRNA gene sequences. The tree was constructed in MEGA 12 using the Tamura-Nei (TN93) model (gamma-distributed rates, shape parameter α = 0.4071). Branch support is based on 1,000 bootstrap replicates, and values ≥50% are shown at the nodes. The scale bar represents 0.10 nucleotide substitutions per site. *Bacillus subtilis subsp. subtilis* strain 168 (PV535807.1) was used as an outgroup. Strains isolated in this study are highlighted in bold; the remaining 15 reference sequences were obtained from the NCBI GenBank database.

**Figure 5:**
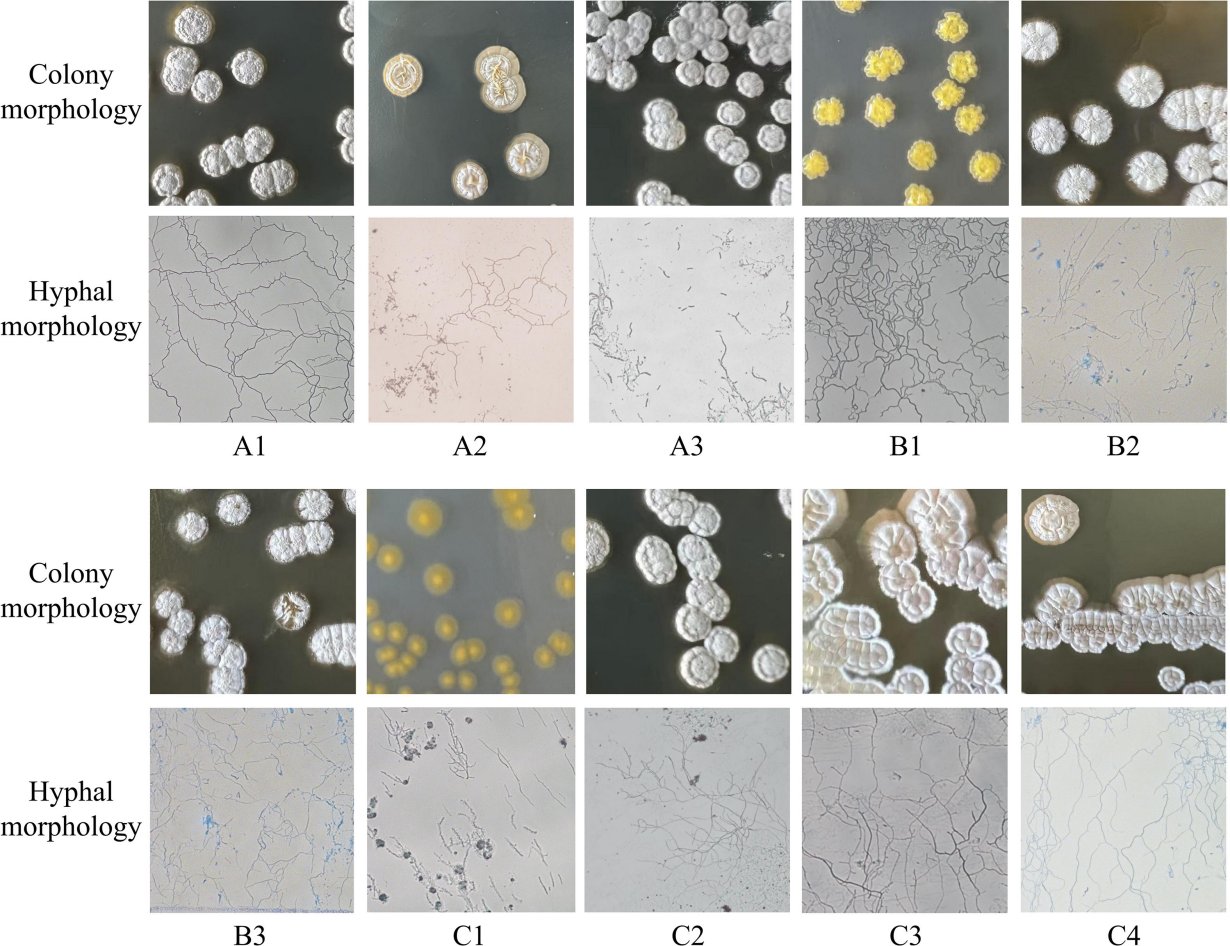
Colonial and mycelial morphology (40×) of 10 Lavender rhizosphere actinobacterial strains. Detailed morphological descriptions for each strain are provided in **Table 1**.

Further screening based on enzymatic activity assays was performed on these 30 strains, from which 10 strains exhibiting superior activity were selected for subsequent experiments. The phylogenetic tree of these 10 strains is shown in **Figure 4**. Morphological observations of the 10 representative strains were carried out on ISP2, ISP3, and ISP5 media (**Table 1**, **Figure 5**) using an inverted microscope (Leica DMI8, Leica Microsystems (Shanghai) Co., Ltd., China). The majority of colonies appeared white to grey-white, powdery, with regular edges and an opaque appearance. Abundant aerial hyphae with branching structures and rich sporulation were observed. All strains were identified as Gram-positive using a Gram staining kit (HuanKai Microbial Technology Co., Ltd., Guangdong, China).

**Table 1:** Colonial morphology characteristics of 10 lavender rhizosphere actinobacterial strains on three culture media (ISP2, ISP3, and ISP5).

Strain number	Characteristics of mycelium and colony morphology
A1	On ISP2, ISP3, and ISP5 media, the colonies are moderate in size, with a powdery white surface, regular edges, and opaque. The mycelium is branched, non-fragmented, and bears conidia at the tips.
A2	On ISP2, ISP3, and ISP5 media, the colonies are large with a smooth, creamy-white surface, irregular edges, and opaque appearance. The mycelium is branched and non-fragmented, with curved aerial hyphae.
A3	On ISP2, ISP3, and ISP5 media, the colonies display moderate size, a gray powdery surface, irregular edges, and an opaque appearance, with observable pigment production.
B1	On ISP2, ISP3, and ISP5 media, the colonies are small in size with a powdery white surface, regular edges, and opaque appearance. Notably, on ISP2 medium, the colonies exhibit a yellow coloration and produce pigment.
B2	On ISP3 and ISP5 media, the colonies are large with a powdery white surface, regular margins, and opaque. On ISP2 medium, colonies appear dark brown with neat edges and are opaque, exhibiting pigment production. The mycelium is branched and non-fragmented.
B3	On ISP2, ISP3, and ISP5 media, the colonies display moderate size, a powdery white surface, regular margins, and an opaque appearance.
C1	On ISP2 and ISP5 media, colonies are small with a powdery white surface, regular margins, and opaque appearance. On ISP3 medium, however, colonies are also small but exhibit a smooth surface, irregular margins, and appear yellow and translucent. The mycelium is branched and non-fragmented, with curved aerial hyphae bearing conidia at the tips.
C2	On ISP2, ISP3, and ISP5 media, the colonies are moderate in size with a powdery white surface, regular margins, and opaque appearance.
C3	On ISP2, ISP3, and ISP5 media, the colonies are large with an uneven surface. The colony margins are powdery white and regular, while the central area is yellow and opaque. The mycelium is branched and non-fragmented, with curved aerial hyphae.
C4	On ISP2, ISP3, and ISP5 media, the colonies are moderate in size with an uneven surface. The colony margins are powdery white and regular, while the central area is yellow and opaque.

### 3.4 Evaluation of salt-alkali tolerance and plant growth-promoting traits in rhizosphere actinobacterial strains

**Figure 6:**
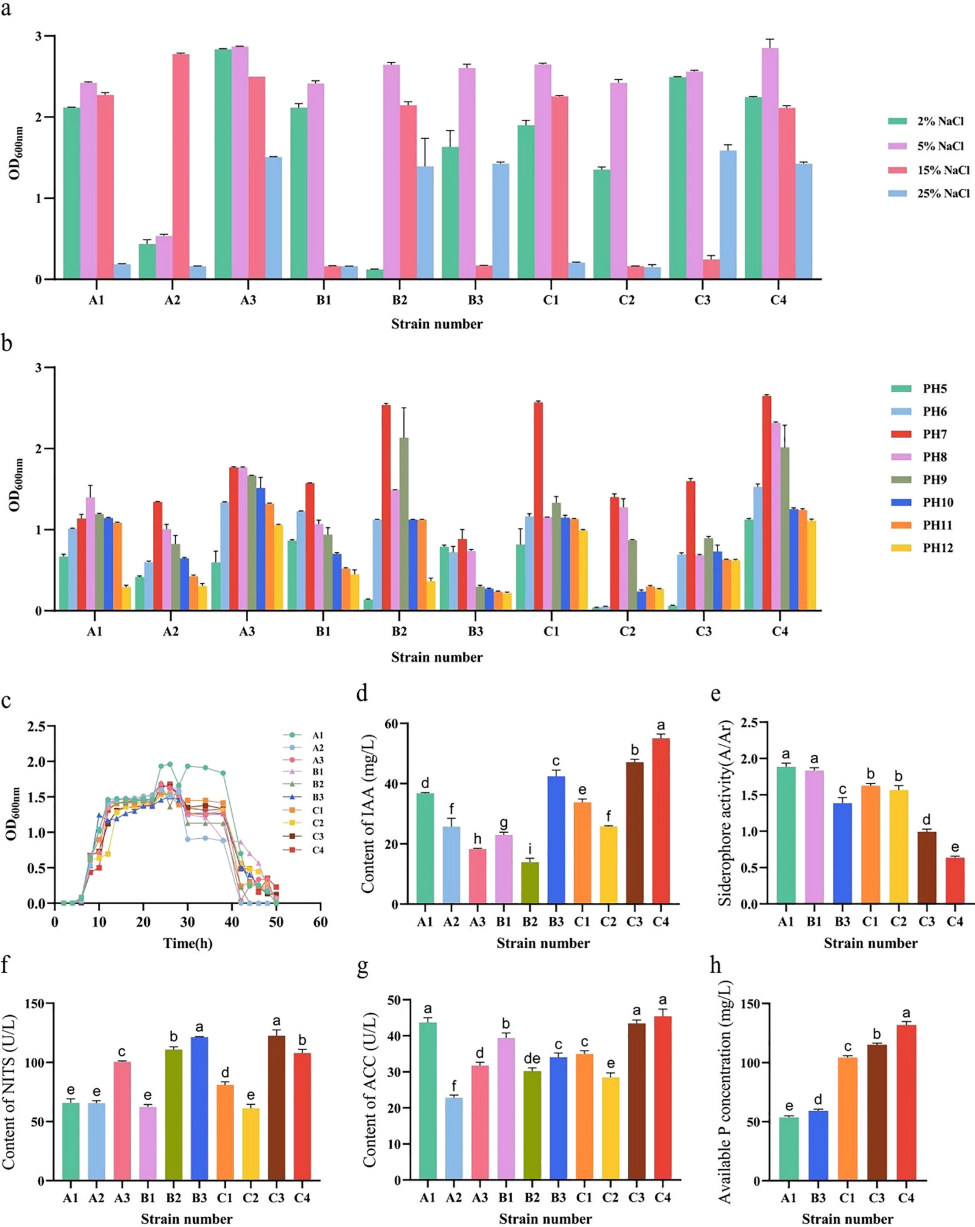
Physiological characterization of salt-alkali tolerance and plant growth-promoting (PGP) traits in selected actinobacterial strains. **(a)** Growth assessment of strains across a gradient of NaCl concentrations (2-25%, w/v). **(b)** Growth assessment across a pH gradient (5-12). **(c)** Growth curves in nitrogen-free Ashby’s medium. **(d-h)** Quantitative evaluation of key PGP traits: **(d)** IAA production, **(e)** siderophore production (expressed as A/Ar ratio; lower values indicate higher production), **(f)** NITS activity, **(g)** ACC deaminase activity, and **(h)** soluble phosphorus content. The results demonstrate that the isolated strains possess broad-spectrum salt-alkali tolerance and multiple functional PGP traits. In **(d-h)**, different lowercase letters indicate significant differences among strains under the same condition (p< 0.05, one-way ANOVA with Duncan’s test, n = 3).

In this study, a systematic evaluation of salt-alkali tolerance and multiple plant growth-promoting (PGP) traits was conducted on ten actinobacterial strains isolated from the lavender rhizosphere soil. The salt and alkali tolerance tests revealed that all strains were capable of growing across a broad range of NaCl concentrations (2%-25%) and pH levels (5-12), demonstrating extensive adaptability to saline and alkaline conditions. Among them, strains A3, B2, and C4 exhibited optimal growth under 5% NaCl, indicating relatively strong salt tolerance (**Figure 6a**). Under highly alkaline conditions (pH 11-12), strains A3, C1, and C4 maintained good growth, suggesting significant alkali tolerance potential (**Figure 6b**).

All tested strains grew in Ashby’s nitrogen-free medium. Growth curves showed that they entered the logarithmic phase at 7 h, reached the peak growth at 24 h, and entered the decline phase after 43 h, preliminarily confirming their nitrogen-fixing potential (**Figure 6c**). Further assessment of indole-3-acetic acid (IAA) production revealed that all ten strains could secrete IAA. After 60 hours of cultivation, the IAA yields ranged between 19.933 and 55.0 mg/L, with strain C4 producing the highest amount, indicating strong phytohormone synthesis capability (**Figure 6d**). Quantitative evaluation of siderophore production showed that strain C4 had the lowest A/Ar value (0.637), indicating the highest relative siderophore yield, whereas strain A1 had the highest A/Ar value (1.887), indicating a relatively lower yield (**Figure 6e**). Measurements of NITS and ACC deaminase activities confirmed that all strains possessed both enzymatic activities. The activity ranges were 22.803-25.409 U/L for NITS and 61.317-122.515 U/L for ACC deaminase. Specifically, the NITS activity of strains B2, B3, and C3 exceeded 110 U/L, and the ACC deaminase activity of strains A1, C3, and C4 exceeded 40 U/L (**Figures 6f, g**). In the evaluation of phosphate solubilization, five strains (A1, B3, C1, C3, and C4) formed solubilization halos on inorganic phosphorus medium. Strain C4 produced the largest halo, and its soluble phosphorus content in liquid culture reached 131.867 mg/L (**Figure 6h**).

These results indicate the successful isolation of a collection of actinobacterial resources from the lavender rhizosphere with broad-spectrum plant growth-promoting potential. Several strains simultaneously exhibited multiple biological functions, including salt-alkali tolerance, enzyme production, nitrogen fixation, phosphate solubilization, siderophore production, and IAA synthesis, demonstrating their potential to alleviate environmental stress and promote plant growth, thereby laying a foundation for their future application.

### 3.5 Evaluating the growth-promoting effects of rhizosphere *Actinobacteria* inoculation in *A*. *thaliana*

**Figure 7:**
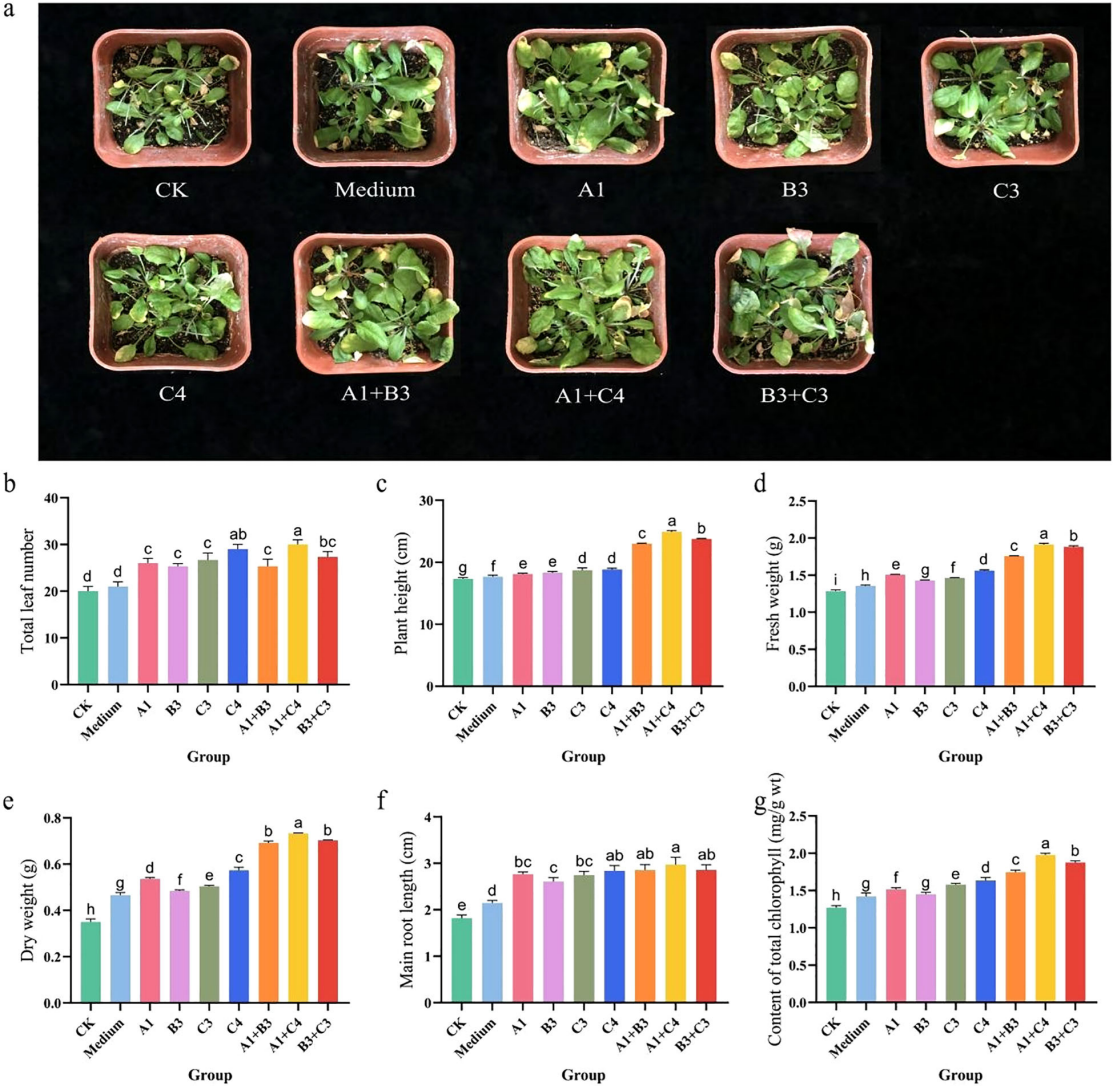
Growth-promoting effects of rhizosphere actinobacterial inoculation on *A*. *thaliana* seedlings. **(a)** Representative phenotypes of *A*. *thaliana* seedlings after 60 days of growth under different inoculation treatments. **(b-g)** Quantitative measurements of growth parameters: **(b)** leaf number per plant, **(c)** plant height, **(d)** fresh weight, **(e)** dry weight, **(f)** primary root length, and **(g)** total chlorophyll content. In **(b-g)**, different lowercase letters indicate significant differences among strains under the same condition (p< 0.05, one-way ANOVA with Duncan’s test, n = 3).

To verify the plant growth-promoting potential of *Actinobacteria* derived from the lavender rhizosphere, four strains (C4, C3, A1, B3) that exhibited excellent performance in prior functional screenings were selected. These strains were prepared as individual inoculants and specific bacterial consortia (C4+A1, C3+B3, A1+B3) and applied to *A*. *thaliana* seedlings via root irrigation. The growth-promoting effects on the plants were systematically evaluated, with the growth status of *A*. *thaliana* seedlings from each treatment group shown in **Figure 7a**.

Among the single-strain treatments, strain C4 demonstrated the most prominent effect. The C4-inoculated plants showed an increase of 9 leaves, a plant height increase of 1.5 cm, fresh and dry weight increases of 0.279 g and 0.224 g respectively, a root length increase of 1.023 cm, and a chlorophyll content increase of 0.366 mg/g wt. The other strains (C3, A1, and B3) also exhibited varying degrees of growth-promoting effects. Notably, the bacterial consortium treatments generally outperformed the single-strain treatments, with the C4+A1 combination showing the most significant effects: plant height increased by 7.587 cm, fresh and dry weights increased by 0.631 g and 0.383 g respectively, and chlorophyll content increased by 0.708 mg/g fresh weight. All these parameters were significantly higher than those of the control group and most single-strain treatments (**Figures 7b–g**).

Additionally, although some parameters in the pure medium treatment group were slightly higher than those in the control (CK), its growth-promoting effect remained significantly lower than all bacterial suspension treatments. This indicates that the observed plant growth promotion primarily resulted from the biological activity of the inoculated *Actinobacteria*, rather than interference from medium components.

In summary, this study demonstrates that *Actinobacteria* originating from the lavender rhizosphere, particularly strains C4 and A1 and their consortium, can effectively promote the growth of *A*. *thaliana* seedlings. They significantly enhance biomass accumulation, root development, and photosynthetic pigment content, demonstrating substantial potential for application as plant growth-promoting bacteria.

## 4 Discussion

This study systematically integrated metagenomic and culturomic strategies to deeply analyze the structure and function of microbial communities in the rhizosphere and endosphere of lavender, successfully isolating multiple rhizosphere actinobacterial strains with significant plant growth-promoting (PGP) potential. The findings not only further confirm the dominant status of *Actinobacteria* in the lavender rhizosphere micro-ecosystem but also provide experimental evidence for their multifaceted mechanisms in regulating plant growth under salt stress.

The composition and function of plant rhizosphere and endophytic microbial communities are highly dependent on plant species, tissue type, and soil environment (Yin et al., 2025). This study found that the diversity and richness of bacterial communities in lavender roots were significantly higher than those in stems and leaves, with the phylum *Actinomycetota* significantly enriched in the roots. This aligns with findings from Yuan et al. (2022) and Park et al. (2023), indicating that plants actively recruit and enrich microbial taxa with specific functions through root exudates, forming niche-specific microenvironments. This view is consistent with that of Bai et al. (2022). As the critical interface for plant-soil microbe interactions, the structure and function of the root microbial community play a vital regulatory role in plant nutrient acquisition, disease resistance, and abiotic stress tolerance.

Further metagenomic functional annotation revealed that the lavender rhizosphere microbiome was enriched with genes associated with carbohydrate metabolism, amino acid metabolism, and the biosynthesis of secondary metabolites. This enrichment was particularly pronounced in the genera *Streptomyces* and *Nocardioides*. These functions are closely linked to plant nutrient uptake, stress response, and the induction of systemic resistance, suggesting that rhizosphere *Actinobacteria* may play a potential role in improving the rhizosphere microenvironment and enhancing plant stress tolerance. For instance, Mendes et al., in their study on disease-suppressive soils, indicated that *Actinomycetota* is one of the most dynamic microbial groups in the rhizosphere, and its abundance is significantly correlated with soil suppressiveness (Mendes et al., 2011). Similarly, Beneduzi et al. systematically summarized how PGPR contribute to plant disease control and growth promotion through mechanisms such as siderophore and antibiotic production, as well as induced systemic resistance (ISR) (Beneduzi et al., 2012). Furthermore, Cordovez et al. revealed that volatile organic compounds (VOCs) produced by *Streptomyces* not only exhibit antifungal activity but also promote plant growth, providing new insights into the chemical communication mechanisms by which rhizosphere microbes regulate plant health (Cordovez et al., 2015). Redundancy Analysis (RDA) results demonstrated significant correlations between key soil physicochemical factors (e.g., pH, available phosphorus, soil organic matter) and the distribution of microbial functions, further corroborating the role of the soil environment in shaping microbial community functionality. This aligns with the conclusions of Mendes et al. regarding the influence of soil properties on microbial community structure (Mendes et al., 2011). Therefore, the *Actinobacteria* enriched in the lavender rhizosphere not only possess broad metabolic potential but also play crucial ecological roles in responding to soil environmental changes, participating in nutrient cycling, and inducing systemic resistance.

At the strain level, we isolated 10 actinobacterial strains with multiple PGP traits from the lavender rhizosphere soil. Most strains possessed capabilities for phosphate solubilization, siderophore production, ACC deaminase activity, and nitrogen fixation, and exhibited good tolerance under saline-alkaline conditions. It is particularly noteworthy that strains such as A1 and C4 showed outstanding performance in IAA synthesis and phosphate solubilization, indicating their dual potential in promoting plant growth and improving nutrient use efficiency. Consistent with studies by Sivagnanam et al. (2022) and Borah et al. (2022) on the potential of *Actinobacteria* in adverse agricultural environments, our findings further validate the promise of *Actinobacteria* as plant growth-promoting resources.

Mechanistically, this study not only verified the direct PGP functions of these *Actinobacteria* but also, through pot experiments with *A*. *thaliana*, revealed their potential in systemically inducing stress resistance. Inoculation with *Actinobacteria*, particularly the C4+A1 consortium, significantly enhanced root length, biomass, and chlorophyll content in plants. This suggests that their growth-promoting and stress-mitigating effects may be achieved through synergistic mechanisms, including the modulation of endogenous hormones, enhancement of antioxidant enzyme activities, and improvement of root architecture. Similarly, *Bacillus subtilis* TCX1 enhanced cucumber resistance to *Fusarium* wilt by producing antimicrobial substances, cell wall-degrading enzymes, and inducing systemic resistance (ISR/SAR), further illustrating the comprehensive advantages of multifunctional microorganisms in plant health management (Dong et al., 2025).

In summary, this study systematically elucidated the ecological functions and agricultural application potential of lavender rhizosphere actinobacteria, spanning from community ecology and functional genomics to strain characteristics and inoculation effects, through an integrated meta-genomics and culturomics approach. The selected elite strains (e.g., A1, C4, and their compound inoculant C4+A1) exhibit both saline-alkaline tolerance and multiple plant growth-promoting traits, providing direct microbial resources and a theoretical foundation for developing specialized microbial agents aimed at mitigating saline-alkali stress and continuous cropping obstacles in lavender cultivation.

It is important to note that the pot experiment in this study employed *A*. *thaliana* as a model plant, with the primary aim of efficiently and controllably providing initial validation of the core plant growth-promoting functions of the screened *Actinobacteria*. While *A. thaliana* offers significant advantages in revealing the fundamental mechanisms of plant-microbe interactions (Uauy et al., 2025), direct extrapolation of these results to lavender or other crops requires caution. Complex factors in field environments (such as climatic fluctuations, soil heterogeneity, competition from indigenous microorganisms, and physiological differences in the crop plants themselves) may significantly influence the colonization efficiency and functional performance of the introduced strains. Future research should advance along two main avenues: on the application front, systematic validation through subsequent pot trials with lavender, followed by field plot experiments and ultimately large-scale demonstrations, is still required to assess the practical value of these strains in real agricultural ecosystems. On the mechanistic front, multi-omics technologies could be employed to decipher the biosynthetic pathways of their key active metabolites, thereby facilitating their precise application in ecological agriculture.

## 5 Conclusions

This study, by integrating metagenomic and culturomic strategies, has elucidated the functional core of the lavender rhizosphere microecology and successfully mined actinobacterial resources that combine saline-alkali tolerance with multiple plant growth-promoting functions. Among these, the compound inoculant C4+A1 demonstrated a significant growth-promoting effect. These elite strains provide a direct resource foundation for developing specialized microbial agents aimed at addressing saline-alkali stress and continuous cropping obstacles in lavender cultivation. The research not only clarifies the ecological functions of rhizosphere *Actinobacteria* but also offers crucial microbial germplasm and a theoretical basis for their practical application in ecological agriculture. It should be emphasized that these growth-promoting effects, observed in the model plant *A*. *thaliana*, require further validation through pot and field trials using lavender as the host plant. Future work should focus on the field efficacy verification and industrial development of these microbial agents.

## Data Availability

The datasets presented in this study can be found in online repositories. The names of the repository/repositories and accession number(s) can be found below: https://www.ncbi.nlm.nih.gov/, ON428493https://www.ncbi.nlm.nih.gov/, ON428494https://www.ncbi.nlm.nih.gov/, ON428495https://www.ncbi.nlm.nih.gov/, ON428496https://www.ncbi.nlm.nih.gov/, ON428497https://www.ncbi.nlm.nih.gov/, ON428498https://www.ncbi.nlm.nih.gov/, ON428499https://www.ncbi.nlm.nih.gov/, ON428500https://www.ncbi.nlm.nih.gov/, ON428501https://www.ncbi.nlm.nih.gov/, ON428502.
